# Optical Imaging of Ventricular Action Potentials in a Torso Tank: A New Platform for Non-Invasive Electrocardiographic Imaging Validation

**DOI:** 10.3389/fphys.2019.00146

**Published:** 2019-02-26

**Authors:** Laura R. Bear, Richard D. Walton, Emma Abell, Yves Coudière, Michel Haissaguerre, Olivier Bernus, Rémi Dubois

**Affiliations:** ^1^IHU Liryc, Electrophysiology and Heart Modeling Institute, Fondation Bordeaux Université, Bordeaux, France; ^2^Univ. Bordeaux, Centre de Recherche Cardio-Thoracique de Bordeaux, U1045, Bordeaux, France; ^3^INSERM, Centre de Recherche Cardio-Thoracique de Bordeaux, U1045, Bordeaux, France; ^4^CARMEN Research Team, INRIA, Talence, France; ^5^CNRS, IMB, UMR 5251, Talence, France; ^6^Bordeaux University Hospital (CHU), Electrophysiology and Ablation Unit, Pessac, France

**Keywords:** ECGI, validation, optical mapping, torso tank, inverse problem

## Abstract

**Background:** Non-invasive electrocardiographic imaging (ECGI) is a promising tool to provide high-resolution panoramic imaging of cardiac electrical activity noninvasively from body surface potential measurements. Current experimental methods for ECGI validation are limited to comparison with unipolar electrograms and the relatively low spatial resolution of cardiac mapping arrays. We aim to develop a novel experimental set up combining a human shaped torso tank with high-resolution optical mapping allowing the validation of ECGI reconstructions.

**Methods:** Langendorff-perfused pig hearts (*n* = 3) were suspended in a human torso-shaped tank, with the left anterior descending artery (LAD) cannulated on a separate perfusion. Electrical signals were recorded from an 108-electrode epicardial sock and 128 electrodes embedded in the tank surface. Simultaneously, optical mapping of the heart was performed through the anterior surface of the tank. Recordings were made in sinus rhythm and ventricular pacing (*n* = 55), with activation and repolarization heterogeneities induced by perfusion of hot and cold solutions as well as Sotalol through the LAD. Fluoroscopy provided 3D cardiac and electrode geometries in the tank that were transformed to the 2D optical mapping window using an optimization algorithm. Epicardial unipolar electrograms were reconstructed from torso potentials using ECGI and validated using optical activation and repolarization maps.

**Results:** The transformation and alignment of the 3D geometries onto the 2D optical mapping window was good with an average correlation of 0.87 ± 0.10 and error of 7.7 ± 3.1 ms with activation derived from the sock. The difference in repolarization times were more substantial (error = 17.4 ± 3.7 ms) although the sock and optical repolarization patterns themselves were very similar (correlation = 0.83 ± 0.13). Validation of ECGI reconstructions revealed ECGI accurately captures the pattern of activation (correlation = 0.79 ± 0.11) and identified regions of late and/or early repolarization during different perfusions through LAD. ECGI also correctly demonstrated gradients in both activation and repolarization, although in some cases these were under or over-estimated or shifted slightly in space.

**Conclusion:** A novel experimental setup has been developed, combining a human-shaped torso tank with optical mapping, which can be effectively used in the validation of ECGI techniques; including the reconstruction of activation and repolarization patterns and gradients.

## Introduction

Non-invasive electrocardiographic imaging (ECGI) is a promising tool to provide high-resolution panoramic imaging of cardiac electrical activity noninvasively from body surface potential measurements (Rudy, 2013). While ECGI has seen considerable development over the past four decades, the inverse problem is ill-posed, highly sensitive to noise, and can have many (both physiological and non-physiological) solutions. As such, a multitude of methods have been, and are still being developed to overcome this (such as regularization) (Tikhonov and Arsenin, 1977; Colli-Franzone et al., 1985a; Wang and Rudy, 2006). Given this ill-posed nature, robust validation is essential for the complete adoption of this powerful technique clinically. Such validation needs to be as close as possible to the conditions of clinical applications, and for applications to arrhythmia, due to their dynamic nature, simultaneous body surface potential mapping (BSPM) and intracardiac recordings is needed.

One of the advantages often cited of ECGI is the ability to map the ventricular surface at a high-resolution, theoretically only limited by the epicardial mesh created. The majority of validation studies to date have evaluated ECGI methods using an *ex vivo* torso tank (Oster et al., 1998; Bear et al., 2018a), *in vivo* large animal models (Oosterhoff et al., 2016; Cluitmans et al., 2017; Bear et al., 2018b), or in patients (Ghanem et al., 2005; Sapp et al., 2012). While there are often several hundred electrodes in the arrays used to provide ground truth data, the spatial resolution (number of electrodes per unit area) is typically lower than that of the cardiac mesh used for ECGI reconstructions. While this means the gross abilities of ECGI to reconstruct cardiac activity can be evaluated, they may not be reliable to assess the accuracy of high-resolution spatial and temporal features such as slow conduction, line of block and/or repolarization gradients. For example, the ability to accurately depict repolarization gradients may be clinically relevant as these have been linked to patients susceptible to ventricular fibrillation (Vijayakumar et al., 2014). No previous study has attempted to validate the accuracy of ECGI to capture gradients in either activation or recovery, possibly because the resolution of ground truth recordings are not high enough.

Currently the only means to validate ECGI at high-resolution is through the use of computational models. This approach has the obvious, significant strength of not requiring the expense and extensive infrastructure of experimental studies, meaning modifying conditions or changing parameters is substantially easier. However, like all data sources, computational models have their limitations including the ability to accurately reflect cardiac pathologies when the ionic mechanisms are unknown and the fact that this approach often commits an “inverse crime” by neglecting the effect of any errors in the problem formulation and using the same problem formulation for both the forward and inverse solutions (Macleod and Brooks, 2000). Validation for ECGI is most ideally performed using an integrative approach with multiple forms of data, and having an experimental or clinical source of high resolution ground truth data would enable this (Cluitmans et al., 2018). The validation of ECGI also depends on its formulation and chosen underlying cardiac source to be reconstructed. The most common formulations use the epicardial extracellular potentials as the cardiac source model (Sapp et al., 2012; Cluitmans et al., 2017; Bear et al., 2018b) which can be compared directly to the signals measured using epicardial mapping techniques. Alternative formulations using transmembrane potential based models, however, require post-processing of the inverse solutions in order to validate against the extracellular potentials measured by an electrode array. The post-processing method chosen may affect the perceived accuracy of the inverse methods used and to date direct validation of reconstructed transmembrane potentials cannot be performed using experimental data.

Optical mapping overcomes the limitations of current ECGI experimental validation setups, enabling high spatial resolution mapping (in the order of 0.5 mm) of cardiac action potentials (Efimov et al., 2004). To date, optical mapping has never been used in the validation of ECGI reconstructions, possibly because the optical mapping window is defined by a 2D surface, and comparing this to ECGI reconstructions based on a 3D cardiac model is a technical challenge. Furthermore, this technique is limited to *ex vivo* models and requires a transparent medium between the camera and cardiac surface.

The aim of this study was to develop a novel experimental set up combining a human shaped torso tank with optical mapping allowing the validation of ECGI reconstructions. The preliminary results presented in this paper aim to demonstrate the application of this setup to validate ECGI reconstructions of activation and repolarization abnormalities and gradients.

## Materials and Methods

This study was carried out in accordance with the recommendations of the Directive 2010/63/EU of the European Parliament on the protection of animals used for scientific purposes and approved by the local ethical committee of Bordeaux CEEA50.

### Experimental Setup

Pigs (*n* = 3; 30–40 kg) were pre-medicated with acepromazine (0.1 mg/kg) and ketamine (20 mg/kg), anaesthetized with propofol (1 mg/kg) and maintained under isofluorane, 2%, in air/O_2_ (50/50) after intratracheal intubation. The thorax was opened and heparin (2 ml) infused intravenously. 1.5 L of blood was collected during intravenous infusion of Voluven. Cardiac arrest was induced with cold cardioplegia introduced into the aortic root. The heart was rapidly excised and immersed in ice-cold Tyrode’s solution. The aorta was cannulated and the heart perfused in Langendorff mode with blood oxygenated with 95/5% O_2/_CO_2_, pH 7.4, temperature 37°C.

**FIGURE 1:**
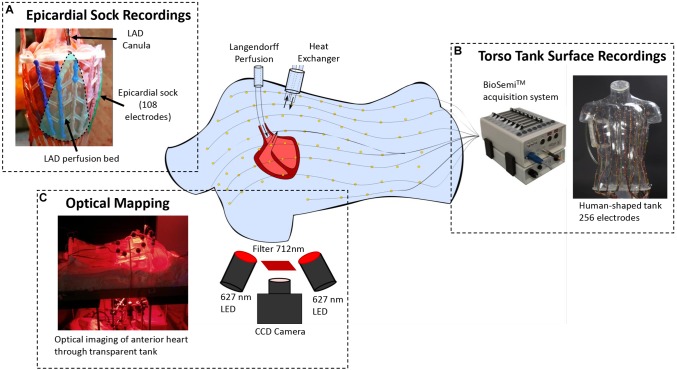
Experimental setup demonstrating **(A)** the pig heart perfused in Langendorff mode with an 108-electrode sock placed over the ventricles and the LAD cannulated on a separate perfusion, **(B)** the human shaped torso tank with 256 electrodes embedded in the surface that the heart is suspended within, and **(C)** the optical mapping that is performed through the anterior surface of the torso.

An epicardial electrode sock (108 electrodes) was attached to the heart (Figure 1A) and bipolar pacing leads were fixed to the right atria (RA) and ventricles with hooks (∼2 mm between electrodes tips). The left anterior descending artery (LAD) was freed above the first diagonal branch for a distance of 5 mm. A cannula was introduced through a small incision, and held in place with a ligature. The cannula was connected to the main perfusion system via a miniature heat exchanger. After instrumentation, perfusion was changed to 100% Tyrode’s solution containing (mM) NaCl, 128; NaHCO_3_, 28; NaH_2_PO_4_, 0.5; MgCl_2_, 0.7; glucose, 11; KCl, 4.7; CaCl_2_, 1.5. The solution was oxygenated with 95/5% O_2/_CO_2_ and kept at pH 7.4, temperature 37°C. The heart was transferred to a human-shaped torso tank made from clear plastic with 256 electrodes embedded in the surface (Figure 1B).

The anterior epicardial surface was imaged using optical mapping through the chest of the torso tank (Figure 1C). Prior to imaging, the heart was mechanically-uncoupled using blebbistatin (15 μM), and stained with the voltage-sensitive dye, Di-4-ANBDQBS (10 μM). The epicardial surface was illuminated with monochromatic LEDs at 627 nm (Cairn Research Ltd., Kent, United Kingdom). Optical images (100 × 100 pixels) of signals passed through a 715 nm long-pass filter were acquired using a Micam Ultima CMOS camera (SciMedia USA Ltd., Costa Mesa, CA, United States) with a spatial resolution of 700 μm/pixel.

### Electrophysiological Recordings

The heart was paced by 2 ms pulses at 2 Hz, with constant current amplitudes 2× the diastolic threshold, on either the left (LV) or right (RV) ventricular epicardial surface, mimicking ectopic activity, and during RA pacing representing normal sinus rhythm. Regional activation and repolarization heterogeneities were introduced through cooling then heating of the LAD perfusate to various temperature (min 21°C; max 40°C), as well as through local perfusion of Sotalol (10 mg/mL) from Sigma-Aldrich (Zwijndrecht, Netherlands). Recordings were taken in these different states during sinus rhythm and ventricular pacing. In total 55 different sequences were obtained across the three hearts.

Electrical and optical signals were measured simultaneously for each sequence. Tank and sock unipolar electrograms were recorded at 2 kHz (BioSemi, Netherlands) and referenced to a Wilson’s central terminal defined using tank electrodes. Optical mapping signals were acquired simultaneously at a frame rate of 1 kHz

### Geometric and Optical Alignment

**FIGURE 2:**
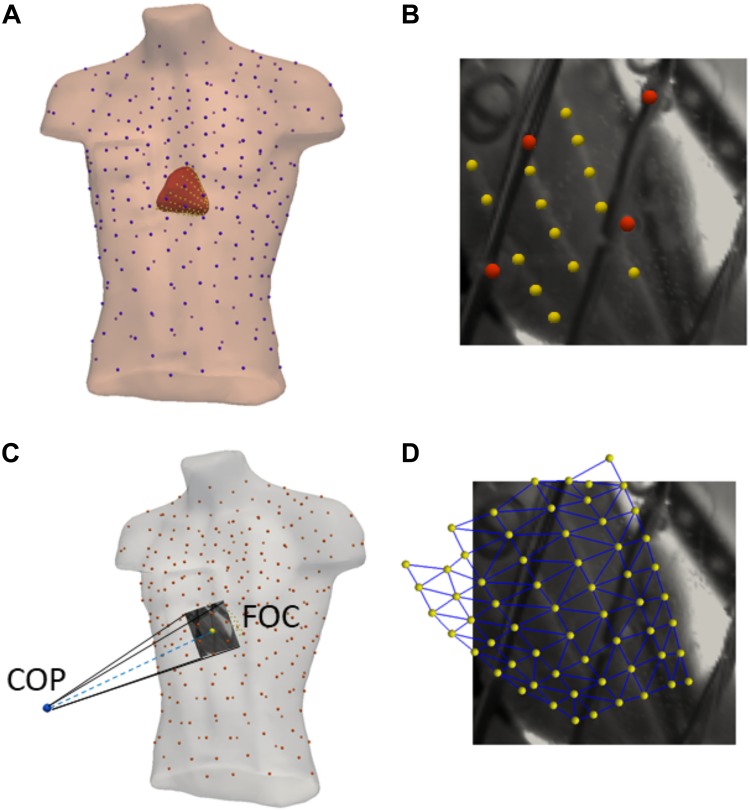
Model creation and optical alignment: **(A)** Meshes are created from 3D angiographic fluoroscopy scans of the epicardial surface (red), sock electrodes (yellow), torso surface (peach), and tank electrodes (blue). **(B)** Example of sock (yellow) and torso (red) electrode identification using the optical mapping field of view. **(C)** Optimization of the camera position (COP) and focal point (FOC) for the perspective projection of the 3D geometries into 2D using the identified sock/torso electrodes. **(D)** Example of the final projection of 3D sock electrodes (yellow) and mesh (blue) onto the 2D optical mapping field of view.

3D angiographic fluoroscopy (Artis, Siemens) was used at the end of each experiment to obtain the exact location and orientation of the epicardium (mean edge length 4 ± 1 mm), perfusion beds and electrodes with respect to the tank (Figure 2A). To align the electrical and optical maps, a perspective projection based on a 3D camera position *X_COP_* and a 3D focal center point *X_FOC_* (Figure 2C) was used to project the torso and sock 3D electrode locations (*E*_3*D*_) onto the 2D optical mapping frames (E^2D) (Figure 2B)

E^2D=E3D⁢ P⁢ (XCOP,XFOP)

Where P is the projection matrix. The camera and focal points were first optimized by minimizing the mean Euclidean distance between projected electrodes positions (E^2D) and their true 2D locations (*E*_2*D*_) visible in optical images (Figure 2C,D).

min⁡XCOP,XFOP⁢ J=min⁡XCOP,XFOPΣ||E^2D−E2D||2n

Where *J* is the cost function and *n* the number of electrodes used in the optimization. This proved to be insufficient due to error in identifying electrodes points in 2D and 3D, therefore a second optimization step was performed minimizing the mean absolute difference in activation times (AT) derived from sock electrodes and optical maps for a single activation sequence:

$$\min_{X_{COP,}X_{FOP}}\,\;J=\frac{{\Sigma |{AT}_{sock}-{AT}_{optical}|}^{2}}{n}$$

To validate the final projection, activation maps (that were not used in the optimization process) derived from sock electrograms and optical mapping signals were compared across all sinus rhythm and pacing sequences (methods described below). The same projection matrix was then applied on the epicardial and LAD geometries, to define their 2D locations in optical mapping window. As the posterior sock electrodes and epicardial surface were not visible in this window, they were removed for all comparisons.

### Signal Processing

Tank and sock channels in which signals were absent as a result of lead fracture or poor electrode contact were immediately evident on visual inspection and were discarded. Electrical signals were temporally aligned to optical maps by a square wave output generated by the optical mapping setup during camera operation that was recorded directly by the BioSemi system. Optical signals were filtered using spatial averaging (kernel 2.1 mm) and temporal averaging (kernel 1.5 ms). A data mask was defined by removing optical signals with an amplitude less than 20% of the maximum signal, removing unconnected components and using a dilation erosion technique to smooth the edges (Laughner et al., 2012). A multi-lead signal averaging algorithm was used to remove remaining noise in both electrical and optical recordings (Aström et al., 2000).

Electrocardiographic imaging electrograms were reconstructed from tank potentials to experiment-specific epicardial surfaces derived from fluoroscopy scans using the method of fundamental solutions (Wang and Rudy, 2006) with Tikhonov regularization (Tikhonov and Arsenin, 1977) and the CRESO method (Colli-Franzone et al., 1985b) to define the regularization parameter.

Activation/depolarization times (AT) were defined from recorded sock electrograms as the time of minimum derivative (dV/dT) of the intrinsic deflection, for optical action potentials as the maximum dF/dT during the action potential upstroke and for ECGI signals by fitting a global activation field to electrogram delays between electrograms (Duchateau et al., 2016). Recovery/repolarization times (RT) were defined from recorded sock and ECGI-derived electrograms as the time of maximum dV/dT of the *T*-wave, a widely used for experimental and clinical electrophysiological studies using unipolar electrograms (Coronel et al., 2009; Lux and Gettes, 2011). For optical signals, repolarization was defined as the time of minimum dF/dT, an index shown to closely match the maximum dV/dT of electrograms (Potse et al., 2009).

### Data Analysis

The minimum dV/dT of recorded electrograms has been shown to very closely match the maximum dV/dT of the action potential upstroke as measured from a floating micro electrode within 1 mm of the small unipolar electrode (Millar et al., 1985; Haws and Lux, 1990). Therefore, transformation and alignment of the 3D geometries into the 2D optical mapping frame were first validated by comparing recorded sock and optical activation maps during sinus rhythm and pacing. As with ATs, a relationship exists between RTs derived from unipolar electrograms and action potentials. RT recorded by sock electrodes were also compared their optical equivalents. Optical activation and repolarization maps were then used to validate ECGI reconstructions. Quantitative comparison of marker timings was performed by defining the nearest optical pixel to each sock electrode and heart mesh node using the Euclidean distance. Activation and RT at these locations directly compared using a root mean square error (RMSE) and Pearson’s correlation coefficient (CC).

Statistical analysis was conducted using GraphPad Prism 7.04. For each metric, the significance of differences was tested using paired *t*-tests with p < 0.05 defined as significant. Data are expressed as mean ± SD unless otherwise stated.

## Results

### Sock and Optical Electrical Alignment

#### Activation Maps

**FIGURE 3:**
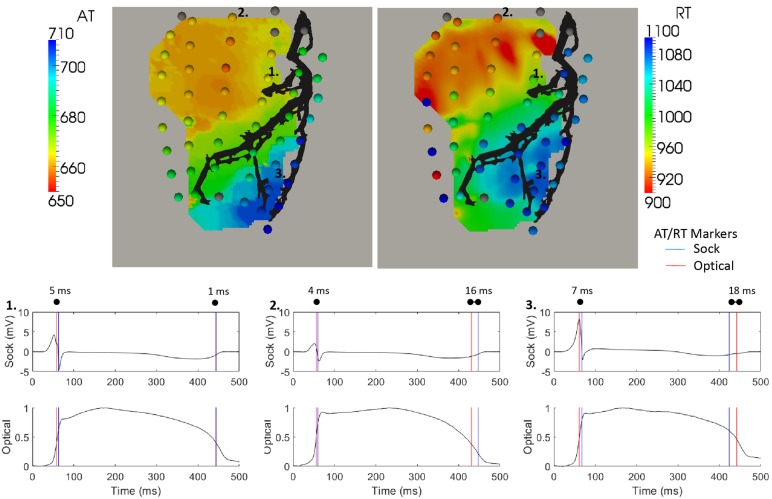
Optical activation (left) and repolarization (right) map with equivalent sock (spheres) timings overlaid during a sinus rhythm sequence with cold perfusion through the LAD (black). Sock electrograms and optical action potentials (normalized) are shown at three electrode locations demonstrating the range in differences between AT and RT markers.

Figure 3 (left) presents an example of an aligned optical and sock activation map during a sinus rhythm sequence with cold perfusion through the LAD marked in black. Sock ATs are represented as spheres with the optical mapping activation underneath. Below, three representative electrograms (top) and action potentials (bottom) are presented with sock (blue) and optical (red) derived AT markers over the QRS.

The pattern of activation derived from sock and optical signals were very similar, as quantified by a correlation of 0.96 and RMSE of 6.1 ms. The larger differences (>5 ms) in ATs were seen when electrograms, action potentials or both had fractionated or shallow intrinsic deflections. This indicates poor electrode contact or fluorescence and naturally making AT calculation more prone to error. The examples in Figure 3 show that despite a 4–7 ms difference in marker placement, the upstroke of the action potential is well aligned with the sock electrograms downslope.

**FIGURE 4:**
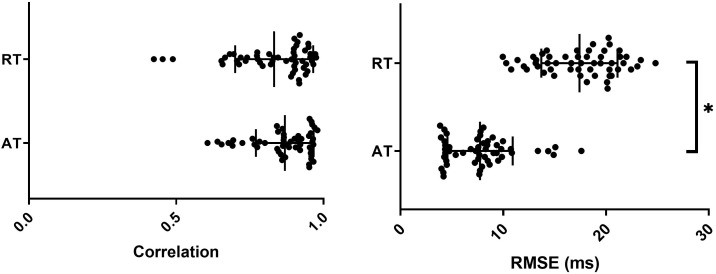
Correlation and RMSE between optical and sock derived activation (AT) and repolarization (RT) times for all sequences with mean and SD overlain. ^∗^represents a significant difference between AT and RT results (*p* < 0.05).

For all experiments, the transformation and alignment of the 3D sock onto the 2D optical mapping window was good as presented in Figure 4, with an average correlation of 0.87 ± 0.10 and RMSE of 7.7 ± 3.1 ms across all activation sequences. There was no significant difference between pacing and sinus rhythm signals for correlation or RMSE (*p* > 0.99).

#### Repolarization Maps

Sock and optical derived repolarization maps were then compared. In Figure 3 (right), the sock and optical repolarization maps are shown for the same sinus rhythm sequence as the activation map (left) described previously. Like activation, the repolarization pattern derived by each technique was the same with late repolarization seen in the LAD perfusion bed. However, the difference in individual RT marker placement were substantially more diverse than with ATs, with differences up to 20 ms. This is seen in the three example electrogram and action potentials presented in Figure 3. The first demonstrates that the negative dF/dT peak in the optical action potentials corresponds very well with the *T*-wave upstrokes in the equivalent electrogram. In second two electrodes, though the *T*-wave upstroke and action potential repolarization curves align temporally, external factors such as poor contact/fluorescence, noise or movement may have substantially shifted marker placement in either or both electrograms and action potentials.

Qualitative comparison of RTs across all sequences demonstrated that sock and optical repolarization patterns were similar, as quantified by high correlation values (0.83 ± 0.13), not significantly different than for ATs (*p* = 0.27). However, the alignment of markers was significantly less accurate than for ATs (*p* < 0.05), with RMSE of 17.4 ± 3.7 (Figure 4). There was no significant difference between experiments (*p* > 0.05), nor between pacing and sinus rhythm signals for correlation or RMSE (*p* > 0.99).

### Optical Mapping for ECGI Validation

#### Activation Times and Conduction Block

**FIGURE 5:**
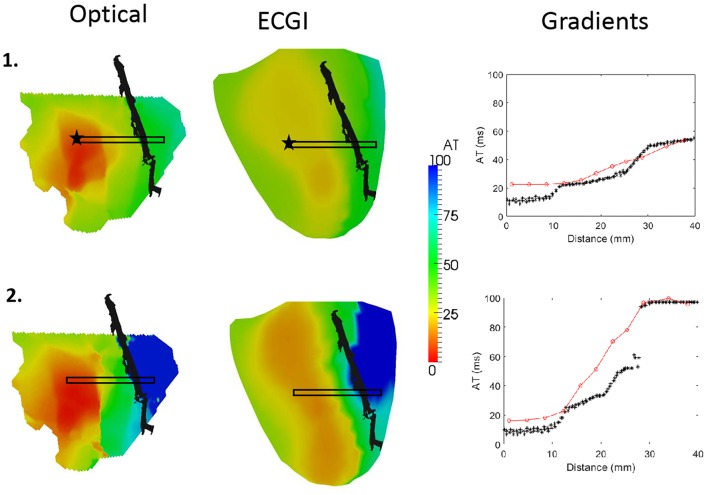
Optical (left) and ECGI (middle) derived activation maps during RV pacing with (1) normal and (2) cold perfusion through the LAD (black). Plots of ATs inside the black box against distance from the star are presented (right) for each sequence derived from optical (black; ^∗^) and ECGI (red; o) signals.

ECGI-derived AT were compared to those derived from the optical action potentials. Figure 5 presents two cases during RV pacing with (1) normal perfusion and (2) cold perfusion through the LAD marked in black. In both cases, ECGI captured the general pattern of activation. However, the timing of the earliest activated region was approximately 15–20 ms after the true onset of activation in both cases as measured with optical mapping. This resulted in a large region of tissue with nearly the same activation time, making it less clear how to define the earliest activation site from ECGI reconstructions.

The advantage of the high-resolution optical mapping is demonstrated by looking at the gradient across the border of the LAD perfusion bed (black box). In the plots on the right we present the ECGI (red) and optical (black) AT within this box against their distance from the black star. In case 2, the cold perfusion creates a line of conduction block across the border of the LAD perfusion bed. ECGI accurately reconstructed the AT on either side of this border, but the conduction block is now seen as a smooth activation wavefront.

**FIGURE 6:**
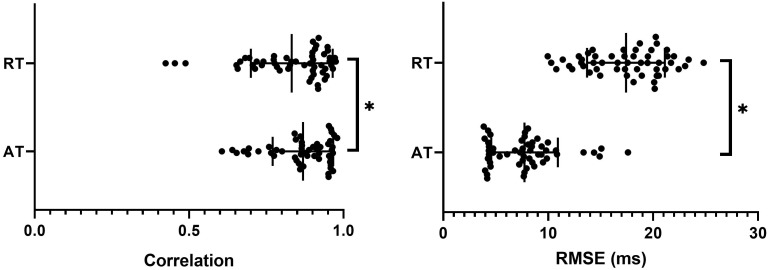
Correlation and RMSE between optical and ECGI derived activation (AT) and repolarization (RT) times with mean and SD overlain. ^∗^Represents a significant difference between AT and RT results (*p* < 0.05).

Quantitative comparison of optical and ECGI activation maps were performed across the entire data set. Using correlation and RMSE, as presented in Figure 6. The majority of cases produced very high correlation (median = 0.83) and low RMSE (1median = 9.6 ms). Qualitative analysis of the three cases with a correlation near 0.5 demonstrated similar activation patterns but shifted slightly in space.

#### Repolarization Times and Gradients

**FIGURE 7:**
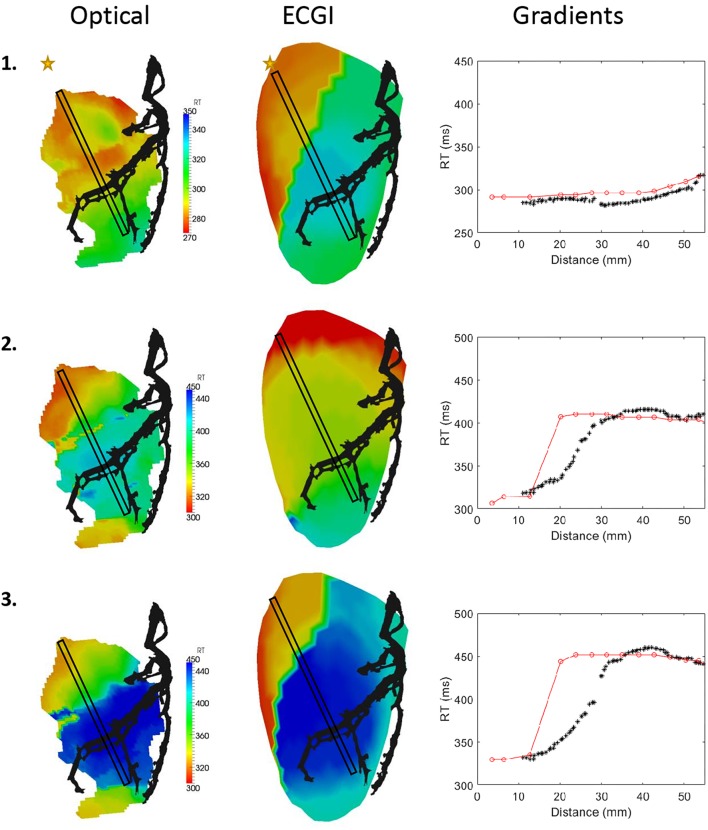
Optical (left) and ECGI (middle) derived repolarizations maps derived from (1) sinus rhythm with normal and (2) cold perfusion through the LAD (black) and (3) LV pacing with cold Tyrode’s and Sotalol perfusion through the LAD. Plots of RTs inside the black box against distance from the star are presented (right) for each sequence derived from optical (black; ^∗^) and ECGI (red; o) signals.

Overall, ECGI did not reconstruct repolarization maps as accurately as ATs, with significantly lower correlation and higher RMSE values (Figure 6). Qualitative comparison of repolarization maps showed that despite this, ECGI accurately identifies regions of late and/or early repolarization during perfusion of hot and cold solution and Sotalol into the LAD. Figure 7 presents three representative examples of optical (left) and ECGI (right) repolarization maps during (1) sinus rhythm, (2) in sinus rhythm with cold perfusion through the LAD (black) to create a gradient in repolarization, and (3) during LV pacing with cold Tyrode’s with Sotalol perfusion through the LAD to augment the gradient. In each example, ECGI clearly distinguishes the regions of early and late repolarization, although the timings are smoothed compared to the optical maps.

The reconstruction of the repolarization gradient across the LAD perfusion bed was assessed by plotting the RTs within the black box against distance from the yellow star (right). In the first case, ECGI accurately shows there is no repolarization gradient. In the second and third cases ECGI captures the existing repolarization gradients though in both cases the gradient is shifted by approximately 10 mm toward the base and is steeper than the ground truth.

## Discussion

This study presents a novel experimental setup combining body surface mapping from a human shaped torso tank with optical mapping of large animal hearts suspended inside. The results of this study have validated the methods for accurately transforming and aligning the 3D epicardial surface onto the 2D optical mapping window. Furthermore, we have demonstrated the setup can be used effectively in the validation of ECGI reconstructed activation and repolarization patterns and gradients.

### Accuracy of Optical Alignment

In order to use the developed experimental setup for ECGI validation, alignment of the 2D optical mapping window with the 3D epicardial mesh derived from fluoroscopy is critical. Many metrics used for ECGI validation are distance-based measurements, where error in the alignment of the “ground truth” would impact the result e.g., localization error of VT exit sites (Wang et al., 2016), premature ventricular contractions (Van Dam et al., 2009), focal discharges and rotor cores (Figuera et al., 2016). In addition, quantitative comparisons of action potentials or activation maps using correlation and RMSE could be dramatically affected by spatial inaccuracies.

In this study, we have developed and validated an optimization method to define the transformation and alignment of the 3D epicardial mesh into the 2D optical window based on markers visible in the both dimensions and one activation pattern derived from recorded sock electrograms and optical mapping (Figure 2).

Comparison of all optical and sock activation maps for each experiment demonstrated that this provides a reliable and robust transformation (Figure 3). On the other hand, repolarization maps showed a larger discrepancy in marker placement between optical and sock signals. As with activation markers, a relationship exists between RT derived from unipolar electrograms and action potentials. However, unlike with ATs this relationship is very sensitive to conditions (Steinhaus, 1989) and has experimentally shown differences in RTs with a standard error from 11 to 26 ms (Wyatt et al., 1981). One explanation for the difference seen here is the fact that the optical signals at the wavelengths used in this study represents an average over a significant depth (up to 4 mm) and lateral distance. Likewise, unipolar electrograms represent the integration of activity through the heart, and particularly the myocardial wall. Although the dF/dt max gives the “true” epicardial activation time [ADD REF to Walton et al. Biophys J 2012], there will still be some blurring, which could play a role in RT marker placement. It is unknown what effects transmural gradients in repolarization play on marker placement for these signals. The large differences seen might also simply be due to the increased inaccuracy in computation of repolarization compared to activation where small movement, ischemia, and signal noise affects the smoothness/duration of the *T*-wave resulting in larger errors in marker placement using automated algorithms. Futhermore, there exists several methods to compute RT from both optical action potentials and unipolar electrograms. While we have chosen indices that have been shown to correlate well from simulation studies (Potse et al., 2009) alternative indices, such as the max d^2^V/dT^2^ typically used for optical mapping signals (Laughner et al., 2012), may result in a closer match.

### Optical Mapping for ECGI Validation

Once the transformation and alignment were defined, ECGI reconstructed activation and repolarization patterns were validated using the optical maps. This is the first study to use optical mapping for the validation of ECGI reconstructions. Several previous studies have evaluated similar ECGI methods using epicardial sock recordings of various resolution to define the ground truth (Bear et al., 2018a,b). In a recent study using the same torso tank model presented here, ECGI was shown to reconstruct activation maps with a correlation of 0.68 ± 0.25 and RMSE = 13.4 ± 5.3 ms in LBBB (Bear et al., 2018a), comparable to the results seen using optical mapping as the reference. The only other studies to report quantitative accuracy of activation maps derived using potential-based ECGI methods have used *in vivo* experimental models. The first in dogs using a 103 non-uniformly spaced electrodes reported a mean correlation values of 0.82 (Cluitmans et al., 2017). The second in pigs using a 239-electrode sock array a mean correlation of 0.78 (Bear et al., 2018b). Despite the differences between these studies in spatial resolution for the ground truth recordings, the results are remarkably similar. This corroboration with previous ECGI validation studies provides further validation of the accurate alignment of the 2D and 3D mapping domains.

Very few studies have evaluated the reconstruction of RT using ECGI, and the only previous experimental study directly comparable to ours in terms of methodology was the study recently performed by Cluitmans et al. (2017) using the *in vivo* dog model. Like activation, despite validation being based on lower resolution ground truth data, the correlation values reported between measured and reconstructed repolarization maps match almost exactly to those seen in this study.

Rather than in the simple comparison of activation and recovery maps, the real benefit of high-resolution optical mapping for ECGI validation is seen in the ability to compare high-resolutions features. Here we have demonstrated one such application in the comparison of activation and repolarization gradients, which has never been previously attempted. It was demonstrated that ECGI reveals the gradients present in both activation and repolarization, although these could be under- and over-estimated, and in some cases shifted slightly in space. For activation maps, underestimation of the gradient may be a reflection of the algorithm used on ECGI signals for marker placement which spatially smooths the activation map. While this typically improves ECGI activation map reconstruction in normal hearts (Duchateau et al., 2016; Bear et al., 2018b), it may also smooth a line of conduction block into a region of slow conduction as seen in Figure 5. In contrast, the over-estimation of recovery gradients seen in Figure 7 is likely due to that same problem that produces artefactual jumps in AT that has previously been noted in ECGI validation studies (Duchateau et al., 2016, 2018). This data and experimental setup will help us to further develop new algorithms to improve activation and repolarization marker detection in the presence of heterogeneities.

In addition to repolarization gradients, this set up will be useful in the validation of ECGI for fibrillation, where an adequate spatial resolution is paramount to the accurate detection of focal sources and rotor cores (Roney et al., 2017); the optimization of parameter selection for ECGI algorithms, such as the choice of regularization parameter; and to evaluate ECGI formulations using transmembrane potentials as the cardiac source, which previously could not be achieved with post-processing of the signals.

### Limitations

The results presented should be considered in light of limitations inherent in the study. First, the field of view of the optical mapping window was limited to approximately 10 cm × 10 cm, only capturing the anterior surface of the heart. While validation of any ECGI algorithm would ideally compare the reconstructions for the entire cardiac surface used, typically the region of interest for high resolution would fit inside this window (e.g., regions of myocardial infarction), and lower resolution mapping of the rest of the heart can be achieved using a traditional electrode sock as has been used in this study.

Another limitation of optical mapping is the necessity of electro-mechanical uncoupling. This removes a condition that is present in clinical applications, and that might impact ECGI reconstruction accuracy particularly during repolarization. However, removing contraction is also an advantage as it enables one to assess the efficacy of ECGI reconstructions without motion artefact. Furthermore, methods are currently being developed to enable optical mapping to counteract motion artefacts, and these methods could be integrated into the system in the future. The use of optical mapping for ECGI validation is limited to using an *ex vivo* model and thus a torso tank with uniform isotropic electrical properties. This is not the case *in vivo* and can result in validation studies reporting better results than are seen using an *in vivo* model. However, like with removing contraction from the heart, torso tank models also allow us to assess the efficacy of a different ECGI formulation when the forward problem is accurately formulated.

Finally, the n number for this study is low, and does not allow insight into inter-heart variability.

## Conclusion

We have demonstrated a novel experimental setup combining BSPM from a human shaped torso tank with optical mapping of large animal hearts suspended inside that can be used in the validation of ECGI reconstructed activation and repolarization patterns and gradients.

## Author Contributions

All the authors contributed to the conception and design of the study, contributed to manuscript revision, read, and approved the submitted version. LB, RW, and EA performed the experimental studies. LB organized the database, performed the statistical analysis, and wrote the first draft of the manuscript.

## Conflict of Interest Statement

The authors declare that the research was conducted in the absence of any commercial or financial relationships that could be construed as a potential conflict of interest.
